# Nurse-Led Interventions to Improve Health, Adherence, and Functional Outcomes in Adults and Older Adults With Multimorbidity: A Systematic Review of Randomized and Quasiexperimental Studies

**DOI:** 10.1155/jonm/6252049

**Published:** 2025-09-19

**Authors:** Suebsarn Ruksakulpiwat, Kewalin Pongsuwun, Pruegsa Junphongsri, Change Preeprem, Sunisa Nguantad, Benjakarn Samart

**Affiliations:** ^1^Department of Medical Nursing, Faculty of Nursing, Mahidol University, Bangkok, Thailand; ^2^Department of Public Health Nursing, Faculty of Nursing, Mahidol University, Bangkok, Thailand; ^3^Department of Nursing Siriraj Hospital, Faculty of Medicine Siriraj Hospital, Mahidol University, Bangkok, Thailand; ^4^Lerdsin Hospital, Bangrak, Bangkok, Thailand

**Keywords:** chronic disease, health outcomes, medication adherence, multimorbidity, nurse-led interventions, systematic review

## Abstract

**Objective:** To synthesize evidence from randomized controlled trials (RCTs) and quasiexperimental studies evaluating the effectiveness of nurse-led interventions on health outcomes in adults and older adults with multimorbidity.

**Background:** Multimorbidity presents complex healthcare challenges and worsened outcomes, especially in older adults. Nurse-led interventions are emerging as a strategic model to address these needs. Evaluating their effectiveness is essential for advancing evidence-based chronic care.

**Design:** A systematic review guided by the Preferred Reporting Items for Systematic Reviews and Meta-Analyses statement.

**Methods:** Two reviewers independently screened studies according to refined inclusion criteria. Risk of bias was assessed using the Risk of Bias 2 tool for RCTs and the ROBINS-I tool for quasiexperimental studies. Data were synthesized using a convergent integrated approach, following the Joanna Briggs Institute methodology.

**Data Sources:** Nursing & Allied Health Collection (ProQuest), PubMed, MEDLINE, ScienceDirect, and Scopus.

**Results:** Thirteen studies met the inclusion criteria. Nurse-led interventions, delivered in person, via telephone, or through digital platforms, were effective in improving care quality, medication adherence, self-management, self-efficacy, and select biophysical indicators such as blood pressure and glucose levels. Some studies also reported reductions in hospital readmissions and mortality. Thematic synthesis revealed four major domains of benefit: health outcomes, self-management and adherence, health functioning, and support and healthcare utilization.

**Conclusion:** Nurse-led interventions contribute to improved health outcomes in adults with multimorbidity by supporting adherence, self-care, and key clinical indicators. These findings highlight the crucial role of nurses in delivering integrated, patient-centered care, supporting their inclusion in chronic disease management strategies.

**Implications for the Profession or Patient Care:** The results highlight nurses' key role in coordinating and delivering effective care. By promoting self-management and adherence, nurse-led models serve as a foundation for managing complex chronic conditions. Broader implementation can improve outcomes and reduce healthcare burdens.

## 1. Introduction

Multimorbidity, the coexistence of two or more chronic health conditions within a single individual, has become a defining challenge in contemporary healthcare systems [1]. Globally, the prevalence of multimorbidity is increasing rapidly due to population aging, lifestyle transitions, and improved survival rates associated with chronic conditions [2–4]. Among older adults, multimorbidity is now the norm rather than the exception, with studies reporting prevalence rates of up to 65% in individuals aged 65 years and older [5]. However, adults under 65 are also significantly affected, particularly those from socioeconomically disadvantaged backgrounds, thereby underscoring the pervasive nature of this phenomenon across age groups [6].

The presence of multiple chronic conditions poses significant risks to physical, emotional, and social well-being [7, 8]. Individuals with multimorbidity are more likely to experience higher rates of hospital readmission, polypharmacy, adverse drug interactions, functional decline, poor mental health, and increased mortality [9–13]. Health systems, traditionally organized around single-disease frameworks, are often ill-equipped to meet the complex and overlapping needs of patients with multimorbidity [14]. Fragmented care pathways, lack of coordination between providers, and insufficient patient engagement further contribute to suboptimal outcomes and increased healthcare costs [15].

Against this backdrop, nurse-led interventions have gained considerable attention as a promising approach to improve care delivery for people with multimorbidity [16]. These interventions are typically characterized by nurses, often in advanced practice or specialist roles, assuming primary responsibility for care coordination, clinical monitoring, health education, lifestyle counseling, symptom management, and follow-up [17]. The strength of nurse-led models lies in their holistic, person-centered philosophy, which aligns closely with the needs of patients with multimorbidity [18]. Furthermore, nurses are uniquely positioned to provide continuity of care, foster patient empowerment, and support self-management through regular contact and trusted therapeutic relationships [19, 20].

Evidence suggests that nurse-led interventions may lead to improvements in a range of outcomes, including better disease control (e.g., blood pressure, glycemic levels) [21, 22], enhanced quality of life (QoL) [23, 24], improved medication adherence [25], reduced hospitalizations [26], and increased patient satisfaction [27]. To the best of our knowledge, no single systematic review has comprehensively examined the core components of nurse-led interventions across diverse patient populations with multimorbidity, nor evaluated their impact on key outcomes such as cost-effectiveness (e.g., reductions in hospital readmissions, shorter lengths of stay, or lower healthcare utilization costs), adherence, and functional health outcomes. Existing reviews are often limited to single conditions, narrow care settings, or restricted outcome domains, reducing their applicability to the broader multimorbid population. Therefore, a systematic review is needed to provide a comprehensive and structured synthesis of the current evidence on nurse-led interventions for adults and older adults with multimorbidity.

To address these gaps, this systematic review aims to synthesize evidence from randomized controlled trials (RCTs) and quasiexperimental studies to evaluate the effectiveness of nurse-led interventions on health, adherence, and functional outcomes in adults and older adults with multimorbidity. The originality of this review lies in its comprehensive analysis of intervention components and their impact across diverse populations and healthcare settings, offering a unified perspective for evidence-based practice. By integrating findings across varied care contexts and outcome domains, this review provides a comprehensive and methodologically rigorous synthesis that will support clinicians in optimizing patient-centered care strategies, inform researchers designing future intervention studies, and guide policymakers seeking evidence-based approaches to improve outcomes and reduce the burden of multimorbidity in healthcare systems.

### 1.1. Objective

To synthesize evidence on the effectiveness of nurse-led interventions in improving health outcomes among adults and older adults with multimorbidity, based on randomized and quasiexperimental studies.

## 2. Materials and Methods

### 2.1. Significance of the Review

Given the growing burden of multimorbidity and the urgent need for scalable, effective models of care, it is essential to synthesize the evidence on nurse-led interventions in a systematic and methodologically rigorous manner. This systematic review aims to fill a critical gap in the literature by examining the effectiveness of nurse-led interventions in improving clinical and patient-centered outcomes among adults and older adults with multimorbidity. By including both RCTs and quasiexperimental studies, this review will capture a comprehensive view of intervention effectiveness across diverse settings and populations.

The results will have practical implications for clinical nursing practice, health systems planning, and health policy. They will also contribute to a growing body of knowledge supporting the development and integration of nurse-led models of care that are responsive to the complexity of multimorbidity. Ultimately, this review will inform the design of future interventions and service delivery innovations aimed at improving health outcomes for one of the most vulnerable patient groups in global healthcare systems.

### 2.2. Design

This study utilized a systematic review design, following the Preferred Reporting Items for Systematic Reviews and Meta-Analyses (PRISMA) guidelines [28], depicting the process of study identification, screening, exclusion, and inclusion.

### 2.3. Search Strategy

A comprehensive literature search was performed across five electronic databases: Nursing & Allied Health Collection (ProQuest), PubMed, MEDLINE, ScienceDirect, and Scopus. The search included studies published from database inception through 31 March 2025 that investigated the effects of nurse-led interventions on improving health outcomes among adults and older adults with multimorbidity. The search strategy combined keywords and subject headings such as “multimorbidity,” “multiple chronic conditions,” “nurse-led,” “nursing intervention,” “randomized controlled trial,” “quasiexperimental,” “health outcomes,” “quality of life,” “hospitalization,” “self-management,” and “medication adherence,” using Boolean operators (AND, OR). In PubMed, medical subject headings (MeSH) such as multimorbidity, nursing care, randomized controlled Trials as topic, and health status were used when appropriate to enhance the precision of the search. In addition, the reference lists of all included articles were manually reviewed to identify any additional relevant studies. All retrieved citations were stored and managed using EndNote X7 for reference management and duplicate removal. The search was limited to published peer-reviewed articles to ensure the inclusion of robust, evidence-based research and to maintain a consistent quality standard across studies.

### 2.4. Study Selection

All retrieved records were screened in a two-stage process. First, titles and abstracts were reviewed to identify potentially eligible studies. Second, full texts of selected articles were assessed against pre-specified eligibility criteria based on the PICOS framework. Two independent reviewers conducted the selection process. Disagreements were resolved through discussion or by consulting a third reviewer. The inclusion criteria ensured alignment with the review objective, while exclusion criteria eliminated studies that did not meet methodological or content-related standards. Full details are provided in Table 1.

### 2.5. Quality Appraisal

To evaluate the methodological rigor of the included studies, we utilized two established tools developed by Cochrane: The Risk of Bias 2 (RoB 2) tool for RCTs and the ROBINS-I (Risk of bias in nonrandomized studies—of interventions) tool for quasiexperimental designs.

For randomized controlled trials, the RoB 2 tool [29] was applied to assess potential bias across five critical areas: the randomization process, adherence to intended interventions, handling of missing data, outcome measurement, and selective reporting. Each domain was rated as having a low risk, some concerns, or a high risk of bias. For nonrandomized studies, the ROBINS-I tool [30] was used to examine bias across seven domains: confounding, participant selection, intervention classification, deviations from intended interventions, missing data, outcome measurement, and selection of reported results. Each study was assigned an overall risk of bias rating of low, moderate, serious, or critical. Two reviewers independently conducted the risk of bias assessments. Any disagreements were resolved through discussion or by consulting a third reviewer to ensure reliability and consensus in the final judgments.

### 2.6. Data Extraction

A structured data extraction form (Supporting Table 1. Summary Table) was developed to ensure consistency, transparency, and completeness in capturing information from each included study. For each study, key bibliographic and contextual information was collected, including the full reference, year of publication, country of origin, and study setting. Population characteristics were recorded, such as the primary chronic disease, coexisting conditions confirming multimorbidity, total sample size, group-specific sizes, mean age with standard deviation, and sex distribution. Study-specific variables included the aim, design (e.g., randomized controlled trial or quasiexperimental), provider(s) involved, and the intervention delivery platform (e.g., in-person, online, or telephone). Detailed information on the intervention was also captured, including the intervention name, content, duration, frequency, and comparator group description. Outcome data encompassed both clinical and patient-reported measures, categorized thematically (e.g., clinical indicators, quality of life, hospital readmission), along with the measurement tools used. Finally, the form documented authors' conclusions, study limitations, and implications for future research.

### 2.7. Data Synthesis

Data from the included studies were synthesized using a convergent integrated approach, following the Joanna Briggs Institute (JBI) methodology for mixed-method systematic reviews [31]. This approach allowed for the integration of diverse quantitative findings by categorizing them into thematic domains. Thematic analysis was used to organize the quantitative findings by identifying common outcomes (e.g., patient satisfaction, self-management skills, adherence, and functional status) and grouping studies that assessed these outcomes for comparison. This method enhanced conceptual clarity and supported a structured narrative synthesis, enabling a comprehensive understanding of the intervention-related themes and their associations with various health outcomes.

## 3. Results

### 3.1. Search Results

**Figure 1:**
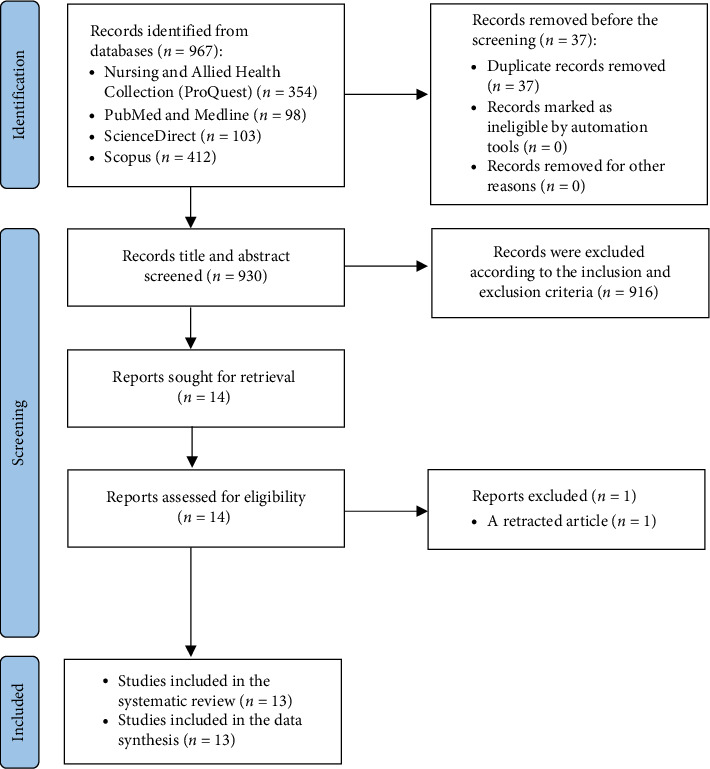
PRISMA Flow Diagram. Note: Adapted from Page MJ, McKenzie JE, Bossuyt PM, Boutron I, Hoffmann TC, Mulrow CD et al. The PRISMA 2020 statement: an updated guideline for reporting systematic reviews. BMJ 2021; 372:n71. doi: 10.1136/bmj.n71 [45].

Following the PRISMA guidelines, a total of 967 records were initially identified through five electronic databases: Nursing & Allied Health Collection (ProQuest) (*n* = 354), PubMed and MEDLINE (*n* = 98), ScienceDirect (*n* = 103), and Scopus (*n* = 412). After removing 37 duplicate records, 930 records remained for title and abstract screening. Of these, 916 records were excluded for not meeting the inclusion criteria. The remaining 14 full-text articles were retrieved and assessed for eligibility. One study was excluded during the full-text screening phase as a retracted article. As a result, 13 studies were included in the final synthesis of this systematic review [32–44]. The study selection process is illustrated in the PRISMA flow diagram (Figure 1).

### 3.2. Description of Included Studies

Table 2 presents the characteristics of the 13 included studies evaluating nurse-led interventions for adults and older adults with multimorbidity. The studies were conducted across 10 countries, with the majority based in high-income settings (*n* = 11, 84.6%). Sample sizes ranged from 30 to 904 participants, and most studies included more than 100 participants (*n* = 10, 76.9%). The mean age of participants ranged from 52.0 to 82.9 years, with the majority of studies (*n* = 10, 76.9%) reporting mean ages above 65 years. Female participants constituted over half of the sample in seven studies (53.8%).

In terms of study design, eight studies (61.5%) were RCTs, including single-blind, cluster, and pragmatic RCTs. The remaining five studies were either quasiexperimental (*n* = 4, 30.8%) or feasibility pre-post designs without a control group (*n* = 1, 7.7%). Multimorbidity was commonly defined as the presence of two or more chronic conditions, including hypertension, diabetes mellitus, chronic heart failure, coronary heart disease, chronic kidney disease (CKD), and chronic obstructive pulmonary disease (COPD). Three studies (23.1%) specifically focused on participants with three or more chronic illnesses.

Interventions were primarily delivered by registered nurses (RNs) or equivalent nursing professionals (*n* = 11, 84.6%), including advanced practice nurses, nurse coordinators, and community nurses. Two studies (15.4%) involved nurse practitioners or mixed teams including nursing students. Intervention delivery platforms varied, with most conducted in-person (*n* = 12, 92.3%), and several incorporating telephone follow-ups (*n* = 6, 46.2%) or digital/telehealth tools (*n* = 2, 15.4%). Intervention durations ranged from 4 weeks to 18 months, with more than half lasting at least 6 months (*n* = 7, 53.8%). Most studies (*n* = 10, 76.9%) compared the nurse-led intervention to usual care, while three studies employed a pre-post or single-group design without a comparator (23.1%).

### 3.3. The Quality Appraisal of the Included Studies

**Figure 2:**
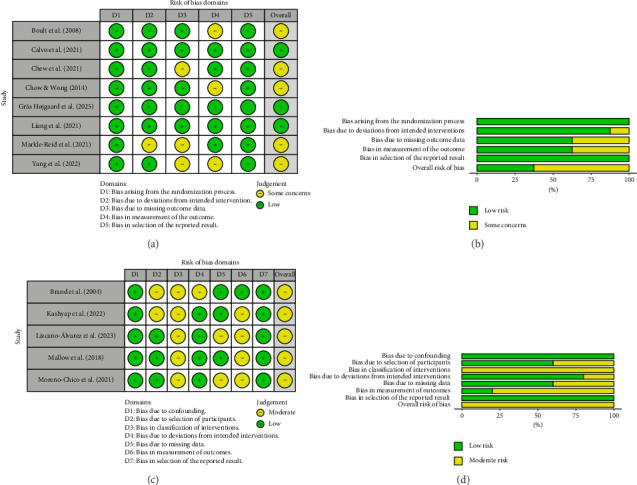
Quality assessment assessed and risk bias. (a) Summary of quality assessment by RoB 2 (*n* = 8). (b) Risk of bias Summary RCTs (*n* = 8). (c) Summary of quality assessment by RoBINS-I (*n* = 5). (d) Risk of bias summary non-RCTs (*n* = 5).

A total of eight RCTs were assessed using the RoB 2 tool, and five quasiexperimental studies were evaluated using the ROBINS-I tool (Figures 2(a) and 2(c)). Among the RCTs, three studies [36, 38, 44] were rated as having a low overall risk of bias across all five domains. The remaining five trials [33, 34, 37, 39, 42] were judged to have some concerns, primarily due to issues related to the measurement of outcomes and handling of missing data. These concerns often arose from the use of self-reported outcome measures or differential loss to follow-up.

In contrast, the five quasiexperimental studies assessed using the ROBINS-I tool were all rated as having a moderate overall risk of bias. All five studies [32, 35, 40, 41, 43] exhibited some concerns in at least three domains, most notably classification of interventions, selection of participants, and measurement of outcomes. The reliance on self-report measures and limited blinding were common sources of bias across these domains.

Bar charts and visualizations (Figures 2(b) and 2(d)) summarize the domain-specific judgments across study types. As shown, RCTs displayed greater consistency and lower risk across most domains, while quasiexperimental studies exhibited a broader range of concerns. All visualizations were generated using the robvis tool [46].

### 3.4. Nurse-Led Intervention Model

Table 3 provides a synthesis of the nurse-led intervention models employed across the 13 included studies. The interventions demonstrate considerable heterogeneity in structure, delivery, and theoretical foundation, reflecting the adaptability of nurse-led models across populations and healthcare systems. Several studies implemented formally named and structured programs such as the Guided Care Model [33], the push–pull–hold (PPH) Program [37], and mI SMART–a mobile health-based chronic illness management program [35]. These models emphasized core principles of continuity, patient engagement, and support for self-management.

Other studies employed unnamed but clearly structured approaches, such as transitional care [32], telehomecare [38], and structured primary care follow-up protocols [43], all of which featured components like health coaching (HC), risk factor monitoring, and personalized goal-setting. Several interventions were theory-informed, including those rooted in self-regulation or activation models, and utilized a range of delivery platforms (e.g., in-person, telephone, or digital technologies).

Overall, the table highlights how nurse-led interventions leverage the holistic, patient-centered strengths of nursing to deliver effective, scalable models of care for individuals with multimorbidity. Despite the variation in nomenclature and structure, common elements included continuity of care, personalized support, and interdisciplinary coordination.

### 3.5. The Effect of Nurse-Led Interventions on Patient Outcomes

**Figure 3:**
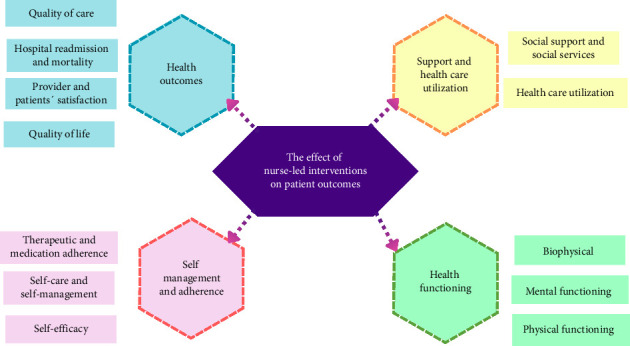
The effect of nurse-led interventions on patient outcomes.

A summary of the findings on the effect of nurse-led interventions on patient outcomes is presented in Table 4 and Figure 3, with additional details provided in Supporting Table 1. Based on the synthesis of 13 studies, 4 major themes were identified: health outcomes, self-management and adherence, health functioning, and support and healthcare utilization, each comprising relevant subthemes. The frequency and distribution of these themes across the included studies are summarized below.

#### 3.5.1. Health Outcomes

##### 3.5.1.1. Quality of Care

Nurse-led interventions were found to significantly improve perceived quality of care in older adults with multimorbidity, as demonstrated in a cluster-randomized controlled trial conducted in urban U.S. primary care clinics [33]. The study evaluated the guided care model, which embedded RNs into primary care teams to provide comprehensive care coordination, including home visits, individualized care planning, and monthly monitoring. Results showed that patients receiving guided care reported significantly higher scores on the patient assessment of chronic illness care (PACIC), particularly in goal-setting (aOR = 2.33, 95% CI: 1.46–3.73), coordination (aOR = 1.87, 95% CI: 1.19–2.93), and decision support (aOR = 1.89, 95% CI: 1.22–2.91). Physicians also reported enhanced satisfaction with chronic illness management. These findings support the role of nurse-led integrated care models in elevating perceptions of care quality among complex patient populations.

##### 3.5.1.2. Provider and Patients' Satisfaction

Kashyap et al. evaluated a nurse-led noncommunicable disease (NCD) clinic implemented in a peri-urban community in India and found uniformly high levels of patient satisfaction. All participants reported being highly satisfied with the services provided, which included screening, counseling using government-developed IEC materials, and regular follow-up. The clinic achieved significant improvements in health indicators such as medication adherence (increasing from 7.8% to 76.4%, *p* < 0.01), blood pressure, blood glucose levels, and risk behavior modification, indicating both the acceptability and effectiveness of nurse-led care [41]. Similarly, Boult et al., through a cluster-randomized controlled trial in the United States, assessed the guided care model among older adults with multimorbidity. Patients in the intervention group reported significantly better experiences in goal-setting (adjusted OR = 2.33), care coordination (adjusted OR = 1.87), and decision support (adjusted OR = 1.89) compared to those receiving usual care. Moreover, primary care physicians involved in the intervention reported increased satisfaction with the management of chronic care, underscoring the model's value in improving both patient and provider experiences [33].

##### 3.5.1.3. Hospital Readmission and Mortality

Six studies [32–34, 36, 38, 40] identified nurse-led interventions as significantly improving hospital readmission and mortality outcomes. For instance, Chow and Wong examined a nurse-led case management program for older adults with comorbidities post-discharge. The intervention included goal setting, health education, home visits, and follow-up calls by nursing students under nurse supervision. Both the home visit and call groups showed significantly lower 84-day hospital readmission rates compared to the control group (*p*=0.018 and *p*=0.007) [34]. Similarly, Liang et al. evaluated a nurse-led tele-homecare program for high-risk patients with multiple chronic illnesses. Participants received vital sign monitoring devices connected to a 24-h nurse call center. Data were collected at baseline and at 3 and 6 months. The intervention significantly reduced mortality (OR = 0.371, *p*=0.027) and emergency department visits (OR = 0.388, *p*=0.013), with longer survival observed in the intervention group [38].

##### 3.5.1.4. QoL

According to five studies [32, 34, 37, 40, 42], nurse-led interventions had a significant positive impact on patients' QoL. To illustrate, Yang et al. evaluated a nurse-led medication self-management intervention for older adults with multimorbidity. Community nurses delivered three educational sessions focused on enhancing knowledge, motivation, and self-management skills, followed by two follow-up support calls. The results showed limited effects on QoL, with significant improvements observed only in satisfaction with medication convenience at visit 1 and reduced medication burden at visit 2. However, no significant effects were found on the overall QoL [42]. In addition, Chow and Wong evaluated a nurse-led case management program designed for older adults with comorbidities following hospital discharge. The program addressed barriers and established shared health goals to enhance self-efficacy and support chronic disease management. The results showed that both intervention groups demonstrated significant improvements in self-rated health, an important indicator of patients' perceived health status and a key component of health-related QoL [34]. These variations in findings may be explained by differences in intervention duration and intensity, as shorter programs with limited follow-up, such as in Yang et al. [42], may not have provided sufficient time for sustained improvement. Moreover, heterogeneity in measurement tools and patient characteristics, including baseline severity of multimorbidity and levels of self-management support, likely contributed to the mixed results across studies.

#### 3.5.2. Self-Management and Adherence

##### 3.5.2.1. Therapeutic and Medication Adherence

Five included studies [36, 40–42, 44] highlight the effectiveness of therapeutic and medication adherence interventions in improving health outcomes among adults and older adults with multimorbidity. For example, a randomized controlled trial by Calvo et al. evaluated the impact of a nursing intervention on therapeutic adherence among elderly patients post-myocardial infarction. At 12 months, adherence was significantly higher in the intervention group compared to the control group (51.9% vs. 21.5%, *p* < 0.001) [36]. Similarly, Yang et al. demonstrated that a 6-week medication self-management program significantly improved adherence immediately following the intervention, indicating a positive short-term effect [42]. Furthermore, a quasiexperimental study by Kashyap et al. (2022) found that medication adherence increased markedly from 7.8% to 76.4% (*p* < 0.01) after 2 months of a nurse-led NCD clinic intervention [41].

##### 3.5.2.2. Self-Care and Self-Management

Five studies [37, 40–43] reported outcomes related to self-care and self-management interventions in improving health outcomes among adults and older adults with multimorbidity. For example, a quasiexperimental study by Lizcano-Álvarez et al. found that intensive and immediate follow-up after myocardial infarction improved compliance behaviors and heart disease self-management. The study suggested that a combined self-care and family care approach may effectively empower post-myocardial infarction patients [43]. Similarly, a two-arm randomized controlled trial by Chew et al. evaluated a novel, theory-driven, nurse-led self-regulation program designed to enhance heart failure self-care behaviors, future thinking, and behavioral automaticity. The intervention group demonstrated significantly greater improvements in self-care compared to the control group, even after adjusting for covariates including gender, living situation, education level, comorbidity, and age [37]. In contrast, a study by Yang et al. assessing the effects of a medication self-management intervention found no statistically significant between-group differences at the 3-month follow-up. There were also no significant differences in participants' beliefs about medication overuse, medication-related social support, or medication skills either immediately after the intervention or at the 3-month follow-up assessment group [42].

##### 3.5.2.3. Self-Efficacy

Three studies [34, 40, 42] demonstrated that nurse-led interventions significantly improved self-efficacy in older adults with multimorbidity. In Chow and Wong's study, a nurse-led case management program incorporating education, follow-up, and goal setting was tailored to barriers related to nutrition, symptom monitoring, and medication adherence. The intervention led to significant improvements in self-rated health and self-efficacy (*F* (2, 277) = 7.72, *p* < 0.001) [34]. Similarly, Yang et al. implemented a nurse-led intervention consisting of three structured educational sessions and two follow-up calls. The sessions addressed medication knowledge gaps, used motivational interviewing to overcome adherence barriers, and developed individualized medication management plans. Participants in the intervention group showed a significant increase in medication self-efficacy (*B* = 1.87, *p*=0.015) [42].

#### 3.5.3. Health Functioning

##### 3.5.3.1. Biophysical

Two studies [35, 41] reported significant biophysical improvements following nurse-led interventions among patients with multimorbidity. Specifically, Kashyap et al. described a nurse-led NCD clinic delivering education, counseling, and referrals over 2 months. After 4 weeks, participants demonstrated significant reductions in systolic (18.75 ± 6.92 mm·Hg) and diastolic blood pressure (4.4 ± 3.71 mmHg), random blood sugar (33.36 ± 38.49 mg/dL), BMI, and waist circumference (*p* < 0.01) [41]. Likewise, Mallow et al. conducted a 12-week intervention using Bluetooth-enabled self-monitoring devices and remote nurse practitioner consultations via the mI SMART platform. This program led to significant decreases in random blood glucose (from 201.93 to 146.79 mmol/L, *p* < 0.001), systolic (134.24–118.93 mmHg, *p* < 0.001) and diastolic blood pressure (88.79–83.62 mmHg, *p* < 0.001), and BMI (36.77–35.05, *p*=0.04) [35].

##### 3.5.3.2. Mental Functioning

Two studies [39, 40] found no significant group differences in mental functioning from baseline to 6 months. In the study by Markle-Reid et al. the mean difference in the mental component score (MCS) from the Veterans Rand 12-item health survey (VR-12) between the intervention and control groups was 1.09 (95% CI: −3.24 to 5.41, *p*=0.61), indicating that the intervention did not have a statistically significant effect on participants' mental functioning over time [39]. Similarly, Moreno-Chico et al. found that although patient activation scores significantly increased at 6 weeks in the HC group compared to the control group (73.29 vs. 66.51, *p*=0.006), this effect was not sustained. There were no significant between-group differences in activation scores at later follow-up points (6 and 12 months), and no significant improvements were observed in secondary mental health outcomes such as anxiety or depression [40].

##### 3.5.3.3. Physical Functioning

One study that addressed physical function, conducted by Markle-Reid et al., showed that no statistically significant group differences were observed in physical functioning from baseline to 6 months. The mean difference between the intervention and usual care groups was −1.45 (95% CI: −4.96 to 2.07, *p*=0.42), indicating that the intervention did not result in a measurable improvement in physical functioning compared to usual care during the study period [39].

#### 3.5.4. Support & Health Care Utilization

##### 3.5.4.1. Social Support and Social Services

Three included studies [39, 40, 42] examined nurse-led interventions on perceived social support and social services, revealing that they could significantly improve social support and social service outcomes. For example, a study by Markle-Reid et al. examined a nurse-led hospital-to-home transitional care intervention (TCI) for older adults with multimorbidity and depressive symptoms. The intervention included goal-setting home visits, telephone follow-up, and a navigation support system by a care transition coordinator (CTC) nurse. The results showed older adults in the intervention group received more information about health and social services (*p*=0.03) compared with the usual care group [39]. However, Moreno-Chico et al. examined a nurse‐led, face‐to‐face, individually tailored HC program for patients aged 18 or older with multiple chronic illnesses. Participants received face‐to‐face, individually tailored HC by a primary care nurse trained as a coach. The intervention showed no significant changes in perceived social support [40]. In addition, Yang et al. evaluated nurse-led interventions on medication-related social support for older people with multimorbidity. The program included in-person follow-up at community health centers (CHCs), along with follow-up via telephone calls. The results showed that no significant changes were found in social support [42].

##### 3.5.4.2. Health Care Utilization

Yang et al. evaluated nurse-led interventions on the utilization of health care services in older people with multimorbidity. This study was a single-blind, two-arm randomized controlled trial conducted in three CHCs in Changsha, China. The intervention consisted of three one-on-one educational sessions on medication-related information, motivation, and self-management skills, and two follow-up phone calls by community nurses, general practitioners, and health care teams. The results showed that no statistically significant effects were found on utilization of health care services at 6 weeks and 3 months' follow-up [42].

## 4. Discussion

### 4.1. Health Outcomes

Across the included studies, nurse-led interventions consistently demonstrated benefits in enhancing perceived quality of care among older adults with multimorbidity. In a cluster-randomized controlled trial, Boult et al. [33] demonstrated that the guided care model, delivered by RNs integrated into primary care teams, led to higher scores on the patient assessment of chronic illness care (PACIC), particularly in goal-setting, care coordination, and decision support. These results align with prior studies showing that nurse-managed care improves the quality of chronic care, especially for patients with complex health needs [47, 48]. The ability of nurses to deliver individualized support, ensure continuity, and coordinate care is a cornerstone of patient-centered care models [47, 49]. These findings highlight the importance of integrating nurses into multidisciplinary teams to deliver structured, coordinated care for people with multimorbidity. Future research should assess the scalability of such models across various healthcare systems, with a focus on long-term outcomes, cost-effectiveness, and equitable access, particularly in underserved populations.

Patient and provider satisfaction also emerged as a recurring theme, reflecting the acceptability and relational strengths of nurse-led interventions. Kashyap et al. [41] found that all patients attending a nurse-led NCD clinic in a peri-urban Indian community expressed high satisfaction with services, which included screening, counseling, and regular follow-up. Similarly, Boult et al. [33] reported that physicians involved in the guided care model also experienced increased satisfaction alongside positive patient outcomes. These findings are consistent with previous research indicating high acceptability of nurse-led care, attributed to enhanced communication and accessibility for patients [50, 51]. Provider satisfaction may reflect reduced workload and improved interprofessional collaboration [52, 53]. The consistent reports of satisfaction across different settings suggest that nurse-led models can enhance care experiences while supporting sustainable chronic care delivery [54]. Future studies should explore the mechanisms contributing to satisfaction and examine the effects of nurse-led models on workforce retention, caregiver burden, and continuity of care.

Notably, nurse-led interventions also demonstrated measurable reductions in hospital readmissions and mortality across multiple settings. For example, Chow and Wong [34] reported significantly lower 84-day readmission rates following nurse-supervised home visits and follow-up calls. Liang et al. [38] found that a nurse-led tele-homecare program, including 24-h remote monitoring, significantly decreased mortality and emergency visits, and improved survival. Faessler et al. [26] further showed that hospitalized patients receiving nurse-led care had fewer readmissions and greater self-care improvements compared to those receiving usual care. Likewise, a randomized trial of a nurse practitioner–led collaborative care model for heart failure demonstrated reduced hospital readmissions [55], and a nurse-led interprofessional team approach was associated with significantly lower all-cause mortality in older heart failure patients with multimorbidity [16]. These findings underscore the value of nurse-led interventions in mitigating adverse outcomes. Future research should evaluate the long-term sustainability of such interventions, identify the most effective components, and explore patient adherence factors to optimize care models for diverse settings.

The evidence on QoL outcomes, however, was more heterogeneous. While Yang et al. [42] found modest improvements in medication-related satisfaction without overall QoL changes, Chow and Wong [34] reported significant improvements in self-rated health following nurse-led case management, a key component of health-related QoL. Supporting evidence from heart failure studies shows that education and follow-up—delivered separately or in combination—improve QoL and self-care [56]. Similarly, Diriba et al. [57] demonstrated that a nurse-led self-management program with family involvement improved both behaviors and QoL in individuals with type 2 diabetes. In addition, a nurse-led multidisciplinary team intervention for atrial fibrillation patients resulted in significantly greater and sustained improvements in QoL compared to usual care at 6 and 12 months [58]. Future studies should assess the long-term impact of various components—such as education, follow-up, family engagement, and team-based care—on QoL to guide the optimization and expansion of nurse-led interventions across settings.

### 4.2. Self-Management and Adherence

A clear pattern emerged across the evidence: nurse-led interventions effectively enhance both therapeutic and medication adherence in adults with multimorbidity [36, 40–42, 44]. Consistent with findings from Berardinelli et al. [25], motivational strategies are commonly employed to promote adherence. Face-to-face nurse-led visits have been shown to significantly improve medication adherence in individuals with chronic conditions. Similarly, Kappes et al. [59] reported that remotely delivered nurse-led interventions not only reduced blood pressure in patients with hypertension but also positively affected cholesterol levels, indicating their potential to enhance overall therapeutic outcomes. Future research should examine the cost-effectiveness of such interventions, particularly in patients with cardiovascular and pulmonary diseases.

Beyond adherence, nurse-led interventions have a pivotal role in fostering self-care and self-management. For example, Subramanian et al. [60] conducted a study in which participants received a video-assisted, nurse-led intervention addressing dietary management, medication adherence, physical exercises, and home care. The program significantly improved self-management in patients with type 2 diabetes mellitus (*t* = 29.639; *p* < 0.001). Similarly, Longhini et al. [61] evaluated transitional care strategies involving follow-up calls, digital platforms, and home visits. Their findings showed that integrated, home-based nursing care improved self-care behaviors in patients with heart failure. Moreover, a meta-analysis by Huang et al. [62] confirmed the efficacy of nurse-led self-care interventions in enhancing self-care among individuals with heart failure. Collectively, these studies underscore the vital role of nurses in promoting health and managing chronic illness. Future nurse-led programs should incorporate psychosocial support to further strengthen self-care and self-management outcomes.

Improvements in self-efficacy were also reported, particularly when interventions integrated education, follow-up, and personalized support [34, 40, 42]. Interventions that combined education, follow-up, and personalized support were particularly effective in building patients' confidence in managing their health. These findings are supported by previous studies, including those by Subramanian et al. [60], which reported similar improvements following nurse-led coaching and counseling among older adults with complex health needs. Future research should identify the most impactful components of nurse-led interventions on self-efficacy and assess their long-term sustainability, cost-effectiveness, and applicability in diverse and resource-limited settings. In addition, evaluating integration into the existing healthcare systems, workforce training, and the effects on caregivers will help optimize implementation and scalability.

### 4.3. Health Functioning

Nurse-led interventions demonstrated consistent improvements in biophysical outcomes, particularly in programs that combined education, monitoring, and individualized support. For example, Kashyap et al. [41] implemented a nurse-led NCD clinic that provided education, counseling, and referrals over 2 months, resulting in notable reductions in blood pressure, blood glucose, BMI, and waist circumference. Similarly, Mallow et al. [35] conducted a 12-week intervention using Bluetooth-enabled self-monitoring devices and remote consultations via the MI SMART platform, which led to significant improvements in blood glucose, blood pressure, and BMI. These findings illustrate the effectiveness of nurse-led strategies that integrate lifestyle education, remote monitoring, and individualized support.

This evidence is consistent with prior research showing that nurse-led care promotes treatment adherence and behavioral change. For instance, Kolcu and Ergun [63] demonstrated improved clinical outcomes through nurse-led hypertension management, while Li et al. [22] highlighted enhanced care continuity via nurse-led chronic care programs. Together, these findings reinforce the critical role of nurses in delivering accessible, personalized care that supports physical health improvement. Future studies should emphasize regular nurse–patient interactions, tailored education, and structured follow-up to strengthen long-term adherence and self-management. Incorporating motivational support, digital tools, and attention to individual and social determinants may further enhance intervention effectiveness. In addition, sustained nurse training and systemic integration are essential for scalability and long-term success.

In contrast, two studies, Markle-Reid et al. [39] and Moreno-Chico et al. [40], reported no significant long-term improvements in mental health outcomes, in spite of using different intervention strategies. While these interventions improved access to information and services, they did not yield measurable changes in mental functioning [39]. Moreno-Chico et al. [40] observed only short-term gains in patient activation, with no sustained effects on anxiety, depression, or perceived support. Conversely, a previous study showed that a nurse-led, multifaceted program in a low-resource setting produced short-term improvements in depression and QoL, although the effects were not sustained [64].

These findings underscore the potential of community-based, holistic interventions that address multiple dimensions of well-being, particularly when tailored to local needs and delivered with a structured follow-up. Improving mental functioning in older or chronically ill populations likely requires long-term, integrative strategies beyond brief coaching or information provision. Future interventions should incorporate ongoing psychological support, behavioral activation, and individualized care planning. Further research is needed to rigorously evaluate which components are most effective and sustainable across diverse populations.

Evidence regarding the impact of nurse-led interventions on physical functioning among older adults remains mixed. Markle-Reid et al. [39] found no significant improvement after 6 months, possibly due to limited duration, insufficient intensity, or a lack of targeted physical rehabilitation. The complexity of the population—older adults with multiple comorbidities—may have also contributed to the limited impact of a generalized intervention. In contrast, a separate study reported that a 12-week person-centered nursing program significantly improved physical outcomes among prefrail older adults, including grip strength, mobility, physical activity, and nutrition [65].

These contrasting findings suggest that the success of physical function interventions depends on multiple interrelated factors, particularly the intervention's duration, focus, and level of personalization. Person-centered, multimodal approaches tailored to the specific limitations and needs of older populations may offer greater potential for improving functional outcomes. Future research should explore how such interventions can be scaled and adapted across diverse healthcare settings to support healthy aging and prevent functional decline.

### 4.4. Support & Health Care Utilization

This review found evidence that nurse-led interventions can significantly enhance perceived social support and access to social services among older adults with multimorbidity. Markle-Reid et al. [39] reported that the community assets supporting transitions (CAST) intervention, delivered by a CTC nurse, improved perceptions of social support. These findings align with Wan et al. [66], who reported increased perceived support among stroke survivors following a peer support intervention.

Nurse-led programs that incorporate peer support, home visits, and telephone follow-ups may enhance perceived social support through several mechanisms. Nurses often provide empathetic listening, psychosocial support, and emotional reassurance, helping patients feel understood and less isolated. They also assist in overcoming barriers to care and coordinate access to essential resources. Importantly, nurses bring a comprehensive and holistic understanding of chronic disease management, which can further strengthen patients' social support networks [67].

However, these positive findings contrast with those of Moreno-Chico et al. [40] and Yang et al. [42], who found no significant improvements in perceived social support following nurse-led interventions. These results are consistent with a meta-analysis by Huang et al. [68], conducted among people living with HIV, which similarly reported limited effects on social support. One possible explanation is that many included studies did not prioritize social support as a primary outcome or design specific intervention components to improve it. This limitation may account for the inconsistent findings. Future studies should explicitly target social support, using intervention designs that include dedicated modules, longer follow-up periods, and more sensitive measurement tools. In addition, larger sample sizes and studies in varied cultural and healthcare settings are needed to more accurately assess the true impact of nurse-led interventions on social support.

This review also found that nurse-led interventions did not demonstrate statistically significant improvements in healthcare utilization among older adults with multimorbidity [42]. Although these interventions showed benefits in medication adherence, self-efficacy, and satisfaction, their effects on reducing healthcare visits, hospital readmissions, or emergency room use were less consistent. In contrast, Li et al. [69] reported that nurse-led TCIs following hospital discharge for heart failure patients were associated with improved healthcare utilization outcomes. Similarly, Chow and Wong [34] found that structured transitional care delivered by nurse case managers significantly reduced healthcare use in older adults with multimorbidity. These results suggest that structured follow-up, individualized case management, and coordinated discharge planning may be critical for achieving reductions in healthcare utilization.

The inconsistent findings may reflect the complexity and severity of multimorbidity in older adults, which often necessitates higher service use regardless of intervention. The effectiveness of nurse-led interventions likely depends on factors such as program intensity, structure, duration, and integration into broader healthcare systems. Interventions that are generic or insufficiently tailored may have a limited impact. Future research should consider healthcare utilization as a primary outcome and adopt study designs that specifically aim to reduce service use. Controlling for patient characteristics, disease severity, and care complexity will help clarify the true effectiveness of nurse-led interventions in this context.

In summary, this review highlights the clear potential of nurse-led interventions to improve patient-centered outcomes such as satisfaction, self-management, adherence, and certain health indicators among adults and older adults with multimorbidity. However, the effects on harder clinical outcomes, including healthcare utilization, physical functioning, and mental health, remain inconsistent. These variations suggest that the effectiveness of nurse-led interventions is highly dependent on their intensity, duration, delivery models, and the degree to which they are tailored to the complex and individualized needs of multimorbid populations. These findings underscore the importance of implementing well-structured, personalized, and integrated nurse-led models of care and evaluating their scalability, cost-effectiveness, and sustainability across diverse healthcare settings.

## 5. Study Limitations

This systematic review has several limitations that warrant consideration. First, the inclusion of only English-language publications may have led to language bias and the exclusion of relevant studies published in other languages, thereby limiting the global applicability of the findings. Second, substantial heterogeneity was observed across the included studies in terms of intervention types, patient characteristics, and outcome measures. This variability hindered direct comparisons and limited the ability to synthesize findings quantitatively, potentially affecting the overall strength and generalizability of the conclusions. Third, most included studies involved participants with a mean age of over 60 years, which may limit the generalizability of our findings to younger adults with multimorbidity. Future research should explore how nurse-led interventions can be adapted and evaluated for younger populations with complex chronic conditions. Fourth, while several RCTs were rated as having a low risk of bias, many studies, particularly quasiexperimental designs, were assessed as having a moderate risk of bias, with common issues including participant selection, classification of interventions, and reliance on self-reported outcomes. These methodological limitations may have influenced the precision and reliability of certain findings and should be considered when interpreting the strength of the evidence and confidence in the conclusions. To address these limitations, future research should employ more standardized study designs, clearly define intervention components and outcome measures, and consider the inclusion of studies published in multiple languages. These steps would enhance the comprehensiveness, comparability, and global relevance of the evidence base.

## 6. Implications for Nursing and Health Policy

The findings of this review underscore the critical role of nurses as leaders within multidisciplinary teams, delivering coordinated, patient-centered care that significantly improves outcomes for individuals with multimorbidity. By integrating clinical expertise with communication and organizational skills, nurses effectively bridge the gap between patients, caregivers, and healthcare providers, ensuring that care plans are comprehensive, personalized, and responsive to the evolving needs of patients. Their leadership not only enhances continuity of care but also facilitates shared decision-making and patient empowerment, which are essential in managing the complex trajectories of multimorbidity. Health policies must urgently support the expansion and long-term sustainability of nurse-led care models across diverse healthcare settings, with a particular emphasis on scalability, equity, and accessibility for underserved populations.

Strategic investments are needed in advanced nursing education, the integration of digital health technologies, and the development of structured follow-up systems. These components are essential for enhancing patient adherence, promoting self-management, and achieving sustainable health improvements. In addition, incorporating psychosocial support into care models is vital for addressing the mental and social dimensions of chronic disease management.

Many nurse-led interventions in this review were delivered as part of multidisciplinary teams that included physicians, pharmacists, and other allied health professionals. These team members contributed specialized expertise in areas such as medication optimization, psychosocial support, and advanced diagnostics, thereby enhancing the comprehensiveness of care. Within these collaborative models, nurses served as the central coordinators, bridging communication among patients, caregivers, and the healthcare team. By monitoring progress, organizing follow-up, and ensuring continuity of care across settings, nurses played a pivotal role in integrating services and tailoring interventions to the complex needs of patients with multimorbidity.

Future health policy frameworks should prioritize rigorous research on the cost-effectiveness, workforce stability, and caregiver implications of nurse-led interventions. Promoting enduring nurse–patient relationships and fully embedding nurse-led models into existing health systems will enhance care quality, reduce hospital readmissions, and strengthen the overall resilience of healthcare systems.

## 7. Conclusion

This systematic review successfully synthesized the available evidence, confirming that nurse-led interventions are effective in improving health outcomes among adults and older adults with multimorbidity. Our synthesis highlights strong evidence for improvements in patient satisfaction, self-management, adherence, and reductions in hospital readmissions and mortality, while noting mixed results for mental functioning, physical functioning, and healthcare utilization. Nurse-led interventions offer a vital approach for managing adults and older adults with multimorbidity by delivering coordinated, patient-centered, and sustainable care. These findings align with our original aim of evaluating the impact of nurse-led models across diverse outcomes, offering evidence to guide clinical practice and health policy. To maximize these benefits, nurse-led models must be expanded across diverse and underserved settings through supportive policies, targeted investments, and ongoing research. Empowering nurses as core members of multidisciplinary teams is essential for advancing equity, efficiency, and the quality of chronic disease management globally.

## Figures and Tables

**Table 1:** Inclusion and exclusion criteria.

**Inclusion criteria**

• Participants aged ≥ 18 years diagnosed with two or more chronic conditions (e.g., diabetes, hypertension, stroke, or CKD), even if the study focuses primarily on one condition. • Nurse-led interventions, operationally defined as structured programs in which a nurse (e.g., registered nurse, advanced practice nurse, nurse practitioner, or nurse specialist) assumes a primary role in planning, delivering, or coordinating care. These interventions may include education, case management, telehealth, follow-up support, self-management coaching, or clinical monitoring, delivered individually, in groups, or via digital platforms (other professionals may be involved in supporting roles). • Comparator: Usual care, standard care, or any intervention not led by nurses. • Outcomes: Health outcomes (e.g., clinical indicators such as blood pressure, HbA1c, hospital readmissions, and mortality), adherence outcomes (e.g., medication adherence, treatment adherence, and self-care behaviors), and functional outcomes (e.g., quality of life, physical functioning, self-efficacy, and patient satisfaction). • Study design: Randomized controlled trials or quasiexperimental studies. • Published in English from inception to 2025.

**Exclusion criteria**

• Studies not involving adults with multimorbidity (i.e., participants with only one chronic disease). • Interventions not led or coordinated by nurses. • Qualitative studies, protocols, pilot studies, or studies without a comparator. • Reviews, commentaries, editorials, letters, or theoretical papers. • Articles not published in English.

**Table 2:** Characteristic of included studies.

References	Country or setting	Research setting	Sample size, total (I/C)	Age (mean ± SD) (I/C)	% female (I/C)	Study design	Conditions	Provider	Platform	Duration	Comparator
[33]	USA	Urban primary care clinics	904 (485/419)	77.2 (NR)/78.1 (NR)	54.2/55.4%	Cluster-randomized controlled trial	Multimorbidity in adults ≥ 65 years	RN and PCPs	In-person	6 months	Usual care
[32]	Australia	Royal Melbourne Hospital (tertiary)	166 (83/83)	77.5 ± 0.81/79.6 ± 1.18	48.2/68.3%	Quasiexperimental	Multimorbidity, chronic heart failure	CDNC	In-person (ward & clinic)	3–6 months	Usual care
[36]	Spain	Tertiary heart institute	143 (68/75)	82.9 ± 5.0/81.6 ± 5.0	44.1/28.0%	Randomized controlled trial	Post-MI with multimorbidity	RN	In-person & telephone	12 months	Usual care
[37]	Singapore	National heart center	144 (72/72)	58.4 ± 14.0/62.8 ± 10.5	25.0/16.7%	Randomized controlled trial	Heart failure with multimorbidity	RN	In-person & telephone	3 months	Usual care
[34]	Hong Kong	Regional hospital	281 (98/87/96)	75.0 ± 8.5/75.5 ± 8.3/77.0 ± 7.6	52.9/54.2/50.0%	Randomized controlled trial	≥ 2 chronic diseases (e.g., HTN, DM)	APNs, nursing students	Home & telephone	4 weeks	Usual care
[44]	Denmark	Community screening clinics	406 (202/204)	67 (NR)	43.6/38.2%	Randomized controlled trial	Screen-detected CVD with comorbidities	RN	Telephone	6 months	Usual care
[41]	India	Public health dispensary	455 (single group)	NR	64.6%	Quasiexperimental	HTN, diabetes, and cancer screening	RN, nursing students	In-person	2 months	No comparator
[38]	Taiwan	Regional hospital	200 (100/100)	80.67 ± 7.29	58%	Randomized controlled trial	Multimorbidity, high readmission risk	Senior RNs	Telehealth + home visits	6 months	Usual care
[43]	Spain	Primary care centers	212 (single group)	80.67 ± 7.29	26.5%	Quasiexperimental (pre-post)	Post-MI CHD	Primary care nurses	In-person	12–18 months	No comparator
[35]	USA	Rural community clinic	30 (single group)	52.0 ± 10.0	70%	Feasibility (pre-post)	HTN, diabetes, and obesity	Nurse practitioners	mHealth (tablet/devices)	12 weeks	No comparator
[39]	Canada	Three academic hospitals	127 (63/64)	77.0 (approximate)	61.7%/63.5%	Pragmatic randomized controlled trial	Multimorbidity + depression	RN and care transition coordinator (CTC)	Home visits + telephone	6 months	Usual care
[40]	Spain	Primary care center	118 (58/60)	64.3 ± 7.86/66.8 ± 7.95	28.8%/28.8%	Quasiexperimental time-series	Multimorbidity (HTN, DM2, COPD, CKD, and rheumatoid)	Primary care nurse trained coach	In-person	6 weeks & follow‐up at 6 & 12 months	Usual care
[42]	China	Community health centers	136 (67/69)	70.76 ± 7.49/72.67 ± 7.64	68.7/52.2%	Randomized controlled trial	Multimorbidity (≥ 3 chronic conditions)	Community nurse	Clinic + telephone	6 weeks	Usual care

*Note:* CVD, Cardiovascular Disease; DM/DM2, Diabetes Mellitus/Type 2 Diabetes Mellitus; HTN, Hypertension.

Abbreviations: APN, advanced practice nurse; CDNC, chronic disease nurse consultant; CHD, coronary heart disease; CKD, chronic kidney disease; COPD, chronic obstructive pulmonary disease; I/C, intervention/control; mHealth, mobile health; NR, not reported; PCPs, primary care physicians; RCT, randomized controlled trial; RN, registered nurse.

**Table 3:** Summary of the nurse-led model.

References	Named model/intervention	Core characteristics
[33]	Guided care model	RN integrates into the PCP team; home visits, care coordination, monthly monitoring, self-management support
[32]	Chronic disease management transitional care service	Nurse-led care transition for high-risk elderly; discharge planning and post-discharge support
[36]	Nurse-led adherence support intervention	Structured education post-MI using checklists and follow-up calls
[37]	Push–pull–hold (PPH) program	Based on temporal self-regulation theory, which includes goal setting, future thinking, and behavior change
[34]	Discharge planning and home follow-up	Three-arm RCT: Hospital discharge + nurse home visit vs. phone follow-up vs. usual care
[44]	Cardiovascular nurse follow-up	Structured telephone follow-up post-screening for AAA/PAD/CP; motivational interviewing techniques used
[41]	NCD risk screening & counseling program	Nurse-led screening and health education focused on BP, BMI, and cancer risk in underserved adults
[38]	Telehomecare program	24 h RN call center + home visits; remote monitoring and early intervention for older adults
[43]	Structured primary care follow-up plan	Nurse-led protocol with 11 visits over 12–18 months; includes risk control and lifestyle coaching
[35]	mI SMART (mHealth intervention for self-management and remote tracking)	Tablet + biometric monitoring devices for chronic illness management, health education, and telecoaching
[39]	Community assets supporting transitions (CAST)	RN transition coordinators, home-based coaching, and mental health support
[40]	Individualized health coaching (HC)	Tailored nurse coaching using activation theory, goal setting, and visual tools
[42]	Multimorbidity management program (MMP)	Community nurse-delivered chronic disease management in primary care with goal setting and lifestyle management

*Note:* PPH, Push–pull–hold program.

Abbreviations: AAA, abdominal aortic aneurysm; BMI, body mass index; BP, blood pressure; CP, carotid plaque; CPP, community partnership program; HC, health coaching; MI, myocardial infarction; MMP, multimorbidity management program; mI SMART, mHealth intervention for self-management and remote tracking; NCD, noncommunicable disease; PAD, peripheral artery disease; PCPs, primary care physicians; RCT, randomized controlled trial; RN, registered nurse.

**Table 4:** The summary of the effect of nurse-led interventions on patient outcomes.

Theme	Health outcomes				Self-management and adherence			Health functioning			Support & health care utilization	
References	Subtheme											
	Quality of care	Provider and patients' satisfaction	Hospital readmission and mortality (e.g., hospitalization, emergency room visits, hospital readmission, general practitioner visits, an and mortality)	Quality of life	Therapeutic and medication adherence	Self-care and self-management (e.g., behavioral automaticity, intention, tobacco and alcohol use, patient activation, medication knowledge, and medication skills)	Self-efficacy	Biophysical (e.g., blood pressure, blood sugar, body mass index, and waist circumference)	Mental functioning	Physical functioning	Social support and social services	Health care utilization
[33]	**X**	**X**	**X**									
[32]			**X**	**X**								
[36]			**X**		**X**							
[37]				**X**		**X**						
[34]			**X**	**X**			**X**					
[44]					**X**							
[41]		**X**			**X**	**X**		**X**				
[46]			**X**									
[43]						**X**						
[35]								**X**				
[39]									**X**	**X**	**X**	
[40]			**X**	**X**	**X**	**X**	**X**		**X**		**X**	
[42]				**X**	**X**	**X**	**X**				**X**	**X**
Number (%)	1 (7.69%)	2 (15.38%)	6 (46.14%)	5 (38.45%)	5 (38.45%)	5 (38.45%)	3 (23.07%)	2 (15.38%)	2 (15.38%)	1 (7.69%)	3 (23.07%)	1 (7.69%)

## Data Availability

The data that support the findings of this study are available in the supporting information of this article.

## Supporting Information

Supporting Table 1. Summary Table.
