# Supplementary material for: Nurse-Led Interventions to Improve Health, Adherence, and Functional Outcomes in Adults and Older Adults With Multimorbidity: A Systematic Review of Randomized and Quasiexperimental Studies
Source: J Nurs Manag. 2025 Sep 19;2025:6252049. doi: 10.1155/jonm/6252049 (PMC12473742; doi:10.1155/jonm/6252049)
Supplement: Supporting Information — Additional supporting information can be found online in the Supporting Information section. [file 6252049.f1.docx]

**Supplementary Table 1.** Summary Table

| **Reference, year, country, setting** | **Primary disease,**  **Chronic conditions included,**  **Sample size (Total, Intervention**  **and control),**  **Age (Mean),**  **Sex (% Female)** | **Study Aim,**  **Study Design,**  **Provider Involved,**  **Intervention Delivery Platform** | **Intervention Detail** | | **Main finding(s)**  **of Included Studies** | **Implications for further research** |
| --- | --- | --- | --- | --- | --- | --- |
|  |  |  | **Intervention group**  **(Duration, Frequency)** | **Control group** |  |  |
| **Ref:** (Boult et al., 2008)  **Year:** 2008  **Country:** USA  **Setting:** Primary care clinics (urban Mid-Atlantic) | **Primary disease:** Not disease-specific  **Chronic conditions included:** Multimorbidity (patients with multiple chronic conditions, aged 65 or older)  **Sample size:**  Total: 904  Intervention: 485 Control:419    **Age in years (Mean±SD):**  Intervention: 77.2 (SD not reported) Control: 78.1 (SD not reported)  **Sex (% Female):**  Intervention: 54.2%  Control: 55.4% | **Study Aim:** To assess the effect of Guided Care on the quality of health care among older adults with multimorbidity  **Study Design:** Cluster-Randomized Controlled Trial  **Provider Involved:** Guided Care Nurse (RN), integrated with Primary Care Physicians (PCPs)  **Intervention Delivery Platform**: In-person (home visits, care coordination across settings) | **Intervention Name:**  The Guided Care model  **Intervention Detail:**  The Guided Care model involved a trained RN embedded in primary care, managing older adults with multimorbidity. Key components included in-home assessment, individualized care planning, monthly monitoring, self-management support, coordination across providers and settings, caregiver support, and linkage to community resources. Nurses collaborated closely with PCPs using electronic health records and team-based communication.  **Duration:** 6 months  **Frequency:** Approximately 2  patients/week per nurse; monthly  follow-ups | Usual care without structured nurse-led intervention | **Primary findings:**   - Patients in the Guided Care group reported significantly higher care quality scores (aOR = 2.03), especially in goal-setting, coordination, and decision support. - Physicians also reported improved satisfaction with chronic care delivery. - Results suggest enhanced patient-perceived care quality and provider experience.   **Other findings:**   - Patients receiving Guided Care reported significantly better care experiences across multiple PACIC subscales, including goal setting (adjusted OR = 2.33, 95% CI: 1.46–3.73) and care coordination (adjusted OR = 1.87, 95% CI: 1.19–2.93). - Improvements were also observed in decision support (adjusted OR = 1.89, 95% CI: 1.22–2.91). Although not all secondary outcomes achieved statistical significance, Guided Care was associated with trends toward enhanced self-management support and continuity of care. - There were no significant differences observed in clinical outcomes such as hospitalization or emergency visits during the 6-month follow-up.   **Measurement of primary finding:** Patient-rated quality of care (PACIC) and Physician Satisfaction Survey | This study highlights the potential of nurse-led Guided Care in enhancing patient-perceived care quality and provider satisfaction among older adults with multimorbidity. However, further research is warranted to evaluate long-term clinical outcomes, healthcare utilization, and cost-effectiveness of this model. Future studies should explore the scalability of Guided Care in diverse healthcare systems and populations, particularly in low-resource or rural settings. Additionally, the impact of such models on caregiver burden, self-management behaviors, and health equity remains underexplored. Rigorous evaluations using standardized outcome measures and extended follow-up periods are essential to support broader policy and practice implementation. |
| **Ref:** (Brand et al., 2004)  **Year:** 2004  **Country:** Australia  **Setting:** Royal Melbourne Hospital, a large tertiary metropolitan teaching hospital | **Primary disease:** Not disease-specific  **Chronic conditions included:** Multiple medical comorbidities (≥2), including chronic heart failure  **Sample size:**  Total: 166  Intervention: 83 Control: 83    **Age in years (Mean±SD):**  Intervention: 77.5 ± 0.81 Control: 79.6 ± 1.18  **Sex (% Female):**  Intervention: 48.2%  Control: 68.3% | **Study Aim:** To determine whether a nurse-led chronic disease transitional care model reduced readmissions to acute care  **Study Design:** Quasi-experimental controlled trial  **Provider Involved:** Chronic Disease Nurse Consultant (CDNC)  **Intervention Delivery Platform**: In-person (pre-discharge ward visit and follow-up clinic visit) | **Intervention Name:** Chronic  Disease Transitional Care Model  **Intervention Detail:**  Pre-discharge assessment and individualized action plan by a CDNC; coordination with the ward team and general practitioner; post-discharge follow-up in a nurse-led clinic providing self-management support, service referrals, and case conferencing. CDNCs were accessible between visits for ongoing patient support.  **Duration:** 3–6 months  **Frequency of Intervention:**  Initial ward visit within 24 hours before discharge; follow-up clinic visits within 2 weeks post-discharge; further contact monthly or as needed. | Usual care is provided by the general medical team, which may include standard discharge planning and outpatient follow-up. | **Primary findings:**   - No statistically significant difference in hospital readmission (adjusted rate ratio: 0.91; 95% CI: 0.59–1.40) or emergency department presentations (rate ratio: 0.90; 95% CI: 0.48–1.70) between groups at 6-month follow-up.   **Other findings:**   - No significant changes were found in quality of life (measured by AQoL), general practitioner visits, or discharge destinations. - Some patients reported individual benefit, but qualitative data revealed persistent system-level barriers, including poor integration, lack of stakeholder engagement, and insufficient resourcing.   **Measurement of primary finding:**  Hospital records (for readmissions and ED visits); AQoL for quality of life. | This study underscores the complexity of evaluating multifaceted transitional care interventions within real-world clinical settings. Although the nurse-led model did not significantly reduce readmissions or emergency visits, future research should examine the interplay between patient-level factors, system constraints, and care integration. Larger, multicenter trials are needed, incorporating patient risk stratification and implementation science methods. Mixed-method evaluations can better illuminate contextual factors and barriers. Long-term outcomes, cost-effectiveness, and sustainability must also be considered to inform broader system-level adoption. |
| **Ref:** (Calvo et al., 2021)  **Year:** 2021    **Country:** Spain    **Setting:** Heart disease institute of a tertiary care hospital. | **Primary disease:** Myocardial infarction    **Chronic conditions included:** Patients aged ≥75 years with myocardial infarction undergoing [percutaneous coronary intervention](https://www.sciencedirect.com/topics/nursing-and-health-professions/percutaneous-coronary-intervention).    **Sample size:**  Total: 143  Intervention: 68  Control: 75    **Age in years (Mean±SD):**  The mean age was 82.2 years.  Intervention: 82.9  Control: 81.6    **Sex (% Female):**  Intervention: 55.9%  Control: 28% | **Study Aim:** To assess the impact of a nursing intervention on therapeutic adherence in elderly patients after myocardial infarction.    **Study Design:** A single-blind, randomized controlled trial.    **Provider Involved:** Registered nurses  **Intervention Delivery Platform**: In-person (education support and patient monitoring) | **Intervention Name:** Nursing intervention    **Intervention Detail:** Two  trained registered nurses  conducted structured interviews  and medication reviews, with  family and caregivers present  when possible. Health  education was subsequently  provided, focusing on clear,  patient-centered  communication, cultural  sensitivity and supportive  strategies to enhance adherence.  A follow-up reminder call at  six months reinforced  adherence and addressed any  ongoing medication concerns.    **Duration:** 3 months    **Frequency of Intervention:** Patients in the intervention group received a 40-minute visit with a registered nurse at three months post-admission, followed by a reminder-type home call at six months. | Usual care management. | **Primary findings:** Therapeutic adherence at 12 months was achieved in a significantly higher proportion of patients from the nursing intervention group (51.9% vs 21.5%, p<0.001).    **Other findings:** No significant differences were observed regarding the proportion of patients requiring readmission at 12 months according to intervention status. Likewise, no significant differences were observed regarding the proportion of patients requiring visits to the emergency room at 12 months.  **Measurement of primary finding:**   - Morisky-Green Levine Medication Adherence Scale - Self-reported compliance communication: Haynes-Sackett method   Records of dispensations in the pharmacy | This study emphasizes that organized nursing interventions led by trained nurses can greatly enhance medication adherence over 12 months in elderly patients with acute myocardial infarction, suggesting valuable clinical, economic, and social outcomes from focused nursing care. Further research should identify effective components of nursing interventions for improving adherence in elderly patients, assess their long-term impact, and examine how individual factors affect outcomes. Standardized adherence measures and replication in diverse settings are essential for strengthening findings and broadening their application. |
| **Ref:** (Chew et al., 2021)  **Year:** 2021    **Country:** Singapore    **Setting:** A tertiary hospital  (Specialist Medical Centre - National Heart Centre Singapore) | **Primary disease:** Heart failure    **Chronic conditions included:**  Patients aged > 21 years old with a clinically diagnosed heart failure for more than one month.    **Sample size:**  Total: 144  Intervention: 72  Control: 72    **Age in years (Mean±SD):**  Total: 60.6±12.5  Intervention: 58.4±14.0  Control: 62.8±10.5    **Sex (% Female):**  Intervention: 25%  Control: 16.7% | **Study Aim:** To examine the effectiveness of a novel theory-driven nurse-led self-regulation program on improving heart failure self-care behaviors, future-thinking, and behavioral automaticity.    **Study Design:** A two-arm randomized controlled trial.    **Provider Involved:** Post-graduate registered nurse experienced in heart failure research.  **Intervention Delivery Platform**: In-person (One face-to-face session, a print booklet) and three reinforcement telephone follow-ups at week 3, 6, and 9. | **Intervention Name:** Push-pull-hold program    **Intervention Detail:** Intervention included one face-to-face session, a printed toolkit, and three reinforcement phone calls. It covered heart failure self-care education, episodic future thinking to boost motivation, goal setting with action planning and self-monitoring, and training in self-regulation and coping strategies. Participants set goals, tracked progress, identified barriers, and applied tailored techniques to manage behavior. The program emphasized future orientation, behavior control, and personal commitment to self-care improvement.  **Duration:** three months  **Frequency of Intervention:** The intervention was delivered through a 30-minute face-to-face session, a printed Push-Pull-Hold Toolkit booklet, and three 10-minute reinforcement phone follow-ups at weeks 3, 6, and 9. | Usual care, including regular outpatient follow-ups with cardiologists to optimize therapy, manage medications, and update treatment plans. Referrals to other healthcare professionals, such as specialists, heart failure nurses, pharmacists, or dietitians, were provided as needed. | **Primary findings:** The intervention group showed significantly greater improvements in heart failure self-care at after a three-month intervention (regression coefficient, B = 13.9, 95% CI: 8.02 to 19.9, p < 0.001) and a further three-month follow-up (B = 8.34, 95% CI: 1.68 to 15.0, p = 0.014).    **Other findings:** No significant differences were found in intention, quality of life, or clinical biomarkers.    **Measurement of primary finding:**   - Self-care of Heart Failure Index (SCHFI) - Consideration of Future Consequences Scale-14 (CFCS-14) - Self-Report Behavioural Automaticity Index (SRBAI)   Minnesota Living with Heart Failure Questionnaire (MLHFQ) | This study punctuates the effectiveness of the Push-Pull-Hold Program in significantly improving heart failure self-care, particularly right after the intervention, attributed to its distinct psychological strategies beyond traditional education. It recommends that future research investigate the distinctions between self-care maintenance and management, evaluate long-term effects using cohort or survival analyses, and examine the program’s relevance across other chronic disease populations. |
| **Ref:** (Chow & Wong, 2014)  **Year:** 2014    **Country:** Hong Kong    **Setting:**  Medical department, general regional hospital | **Primary disease:** Not disease-specific  **Chronic conditions included:** Co-morbidities (older patients of age >65, having at least two chronic diseases, including: chronic respiratory, cardiac, type 2 diabetes, and renal diseases)    **Sample size:**  Total: 281  Intervention  - Home visit: 87  - Call: 96  Control: 98    **Age in years (Median (range):**  Total: 76 (60-92)  Intervention  - Home visit: 75 (60-92)  - Call: 75·5 (60-89)  Control: 77 (60-89)  **Sex (% Female):**  Total: 52.3%  Intervention  - Home visit: 52.9%  - Call: 54.2%  Control: 50% | **Study Aim:** To examine the effects of a nurse-led case management program for hospital-discharged older adults with co-morbidities.    **Study Design:** Randomized controlled trial    **Provider Involved:**   - Case manager: Nursing practitioners with over 15 years of experience in medical nursing and discharge planning at the study hospital. - Senior-year nursing students were recruited to assist in part of the interventions.   **Intervention Delivery Platform**: In-person (home visit group), telephone (call group) | **Intervention Name:** Nurse  case management intervention    **Intervention Detail:** The nurse case management (NCM) intervention identified barriers and set mutual goals with patients to help them manage health activities such as nutrition, symptom monitoring, and medication adherence, aiming to improve self-efficacy and support chronic disease self-management. The NCM coordinated interventions, including home visits and phone calls made by senior nursing students. For the home visit group, the NCM conducted the first visit, followed by a second visit by nursing students and a final call in the fourth week. In the call group, nursing students made two calls post-discharge, with NCM support as needed.    **Duration:** 4 weeks  **Frequency of Intervention:** once a week for four consecutive weeks. | Research assistants made two social calls to patients within 4 weeks, focusing on topics like the weather, TV programs, or leisure activities, without including self-management content. Each call lasted about 5 minutes. If patients had health-related questions, research assistants advised them to contact healthcare professionals using the provided contact numbers. | **Primary findings:** The interventions showed notable differences in hospital readmission rates within 84 days after discharge (x^2^ = 8.03, p = 0.018), with the two intervention groups experiencing lower readmission rates compared to the control group (x^2^ = 7.25, p = 0.007).    **Other findings:** The patients in both study groups reported significantly better self-rated health and self-efficacy (F _(2, 277)_ =7.72, p < 0.001).    **Measurement of primary finding:** The data for 28- and 84-day readmissions were retrieved from the hospital's administrative record system. | This study demonstrates that nurse-led, individualized, and collaborative interventions significantly reduced hospital readmissions and improved self-rated health, self-efficacy, and physical health among older patients with comorbidities. Despite differences in delivery platforms, their success largely depended on the quality of patient-nurse interactions. Future research should investigate the specific mechanisms through which these interventions improve outcomes, with particular attention to the role of patient-nurse relationships. Comparative studies on delivery methods (e.g., in-person vs. telehealth), assessments of long-term impacts on readmissions, self-efficacy, and quality of life, as well as evaluations of scalability and cost-effectiveness across diverse healthcare settings, are also recommended. |
| **Ref:** (Gräs Højgaard et al., 2025)  **Country:** Denmark  **Setting:**  Community screening clinics | **Primary disease:**  cardiovascular disease (CVD)    **Chronic conditions included:** AAA, PAD, CP, hypertension, Type 2 diabetes, arrhythmia, and cardiac ischemia.  **Sample size:**  Total: 406  Intervention: 202  Control: 204    **Age in years (Mean ± SD):**  aged 67 years    **Sex:** females 40.9%, males 59.1%  Intervention F:43.6%  Control F: 38.2% | **Study Aim:** To investigate the effect of nurse-led telephone follow-up (TFU) on medication adherence after screen-detected cardiovascular disease (CVD)    **Study design:** randomized controlled trial    **Provider involved:** registered screening nurses    **Intervention delivery platform:** telephone | **Intervention Name:**  telephone follow-up (TFU) on medication adherence after screen-detected cardiovascular disease (CVD).  (TFU intervention)    **Intervention Detail:**  -The nurse-led intervention consisted of three TFUs conducted at 1, 3, and 6 months after the recommended initiation of preventive medication due to screen-detected CP, PAD, and/or AAA  -Motivational interviewing (MI)  techniques were also incorporated to support participants in making informed decisions about initiating preventive CVD medication. -Motivational interviewing focuses on improving health behaviours, like medication adherence, by enhancing  motivation and change commitment. It emphasizes understanding participants’ health perspectives, respecting autonomy, building confidence, and supporting  decision-making  **Duration:** 6 months  **Frequency:**  conducted at 1, 3, and 6 months after the recommended initiation of preventive medication due to screen-detected CP, PAD, and/or AAA | Usual care. | **Primary findings:**  The primary outcome was medication adherence after 1 year.  -no intervention effect for lipid-  lowering medication [odds ratio (OR): 1.06, 95% confidence interval (CI): 0.69–1.63, P = 0.800] or anti-platelets (OR:  0.93, 95% CI: 0.62–1.39, P = 0.732).    **Other findings:**  Secondary outcomes were quarterly  point prevalence time after the recommendation. | Future studies should focus on overcoming barriers to CVD medication adherence by customizing interventions to individual citizens’ adherence barriers and elucidating intervention mechanisms. Further research may focus on the development of medication decision-making tools, using adherence may require more than a phone call. |
| **Ref:** (Kashyap et al., 2022)  **Country:** India  **Setting:**  NCD clinic,  Public health  Dispensary Sector-25 Chandigarh. | **Primary disease:** Not disease-specific    **Chronic conditions included:** HT, DM, and CA screening    **Sample size:** 455    **Age:** ≥30 years  The mean age is not seen    **Sex:** female 64.6% | **Study Aim:** to assess the feasibility and effectiveness of a nurse‑led NCD clinic for identification,  prevention, and management of common NCDs.    **Study design:**  A quasi-experimental study.    **Provider involved:**  nurse supervisors, nursing students.  **Intervention delivery platform:** In-person. | **Intervention Name:**  Task sharing for managing  common noncommunicable  diseases in a nurse-led  noncommunicable disease  clinic in the Peri‑Urban \  Community of Chandigarh    **Intervention Detail:**  -Nurses were trained for a  period of 6–8 hours by the  nurse supervisors. The  nurse‑led NCD clinic was set  up in a separate room and ran for over 2 months.  -The primary outcome of the  study was the proportion of the  population screened, prevalence  of common NCDs, risk factors  modification, and medication  adherence.  -An interview schedule was used to collect sociodemographic details and clinical profiles of patients.  -After screening, patients were referred to the medical officer for prescription if required. Education and counseling for risk factors modification (government of India IEC material) by using colored pamphlets, flash cards, sharing the video, and a PDF to study participants who have an Android phone. Follow‑up for patients was done after 4 weeks to assess the medication adherence and risk factors modification.  **Duration:** 2 months  **Frequency:**  Nurses were trained for a period of 6–8 hours. The frequency of the clinic was 6 days a week. | Usual care | **Primary finding:**  -Proportion of population in better medication adherence group increased from 7.8% to 76.4%.  Significant improvement was also seen in tobacco and alcohol use, with a quit rate of 21.7% and 16%, respectively. All the study participants were highly satisfied with the nurse-led NCD clinic.    -There was a significant mean change in systolic blood pressure (18.75 ± 6.92 mm Hg), diastolic blood pressure (4.4 ± 3.71 mm Hg), random blood sugar (33.36 ± 38.49 mg/dl), Body Mass Index,  and waist circumference (P < 0.01) among the population screened. Medication adherence significantly increased from 7.8% to 76.4% (P <  0.01) after 2 months of nurse‑led NCD clinic. | The study underscores nurses’ key role in preventing and managing non-communicable diseases. Future research should evaluate the long-term effectiveness, sustainability, scalability, cost-effectiveness, and impact of nurse-led NCD clinics across diverse healthcare settings. |
| **Ref:** (Liang et al., 2021)  **Country:**  Taiwan    **Setting:**  Regional hospital | **Primary disease:** common NCDs  **Chronic conditions included:** Multimorbidity  (age > 65 years, high risk  for readmission with a length of stay, acuity of admission, comorbidity, and visits to ED (LACE) index of ≥7)    **Sample size:** 200  **intervention group** 100 **control group** 100.    **Age:**  mean age was 80.67 years  (SD = 7.29)    **Sex:** women 116 (58.0%) | **Study Aim:** to evaluate  the effectiveness of an integrated nurse-led tele-homecare program for patients with multiple chronic illnesses and a high risk for readmission.    **Study design**: A randomized controlled trial.    **Provider involved:**  senior nurses, physicians, and technology engineers.    **Intervention delivery platform:** Telehealth and home visits | **Intervention Name:**  Tele-Homecare program for patients with multiple chronic illnesses and a high risk for readmission.    **Intervention Detail:**  For each participant in the IG,  provided wireless transmission, devices, including a one-touch smartphone, blood pressure (BP) monitor, medication dispenser, and a necklace emergency call button. a glucometer to measure their blood sugar.  -The one-touch smartphone provided to the patient or caregiver enabled communication with a nurse-led 24-hr call center, and the smartphone was used to set up twice daily reminders to check vital signs  -The one-touch smartphone then were automatically transmitted to the 24-hr call center and healthcare record system via Bluetooth to assist in physician assessment. -Each participant checked their biological  parameters twice a day (at 8:00 a.m. and 6:00 p.m.).  -Data were collected at three time points: pretest baseline (T0), 3 months after intervention (T3), and 6 months after the intervention.  -A generalized estimating equation model was used to compare changes and evaluate the effect of differences between the two groups over time.    **Duration:** 6 months    **Frequency:**  -Three face-to-face interviews were conducted on the day of discharge from the hospital (T0), after 3 months (T3), and after 6 months (T6). | **Control group**  team members included four senior nurses and physicians. Before discharge (T0), patients received  discharge planning to promote their home care ability (content of care included assessment, nutrition and  medication consultation, and medication reminders).  The home visits were made by the nurses at the patients’ homes for provided home care (content of  care included assessment, checking vital signs, patient education, nutrition and medication consultation, and  medication reminders)  and data collection at 3 months  after discharge (T3) and 6 months after discharge | **Primary outcome:**  -found that the tele-homecare program significantly reduced mortality and ED visits, whereas no significant effect on readmission was observed**.**  -eight patients (8%) in the IG and 19 patients (19%) in the CG were deceased, and the difference was significant (odds ratio [OR] = 0.371, 95% CI = 0.154–0.892, p = .027).  -The Kaplan–Meier survival analysis of time to death indicated that patients in the IG had a significantly longer survival time than those in the CG.  -The readmission rate was 44% in the IG and 41% in the CG, and the difference was not significant (OR = 1.131, 95% CI = 0.645–1.981, p = .668).  -Twelve percent and twenty of IG and CG patients, respectively, had ED visits, and the difference  was significant (OR = 0.388, 95% CI = 0.183–0.822, p = .013).    **Secondary outcome:**  -patients’ QOL indicated significant improvement.  - The mean medication adherence scores in  the CG and IG revealed a stable status from T0 to  T6. Medication adherence scores in the IG slightly increased from T0 to T3 and T6. However, no significant interaction effect was noted in the GEE model at T3 (β = 0.636, p = .130) and T6 (β = -0.082, p = .771). This finding indicated that the tele-homecare program could not effectively enhance patients’ medication adherence. | Tele-homecare programs may reduce mortality and emergency visits, improving patient outcomes. Further research is needed to understand how these benefits occur and to identify which patient groups gain the most, enabling tailored tele-homecare plans. |
| **Ref:** (Lizcano-Álvarez et al., 2023)  **Year**: 2023  **Country**: Spain  **Setting**: 40 primary care facilities in Madrid, Spain | **Primary disease:** Acute Coronary Syndrome  **Chronic conditions included:** HT, DM, DLP, Obesity, Smoking history  **Sample size:** Total 212 patients. Completed intervention on 132 patients  No control group.  **Age (Mean):** -  **Sex:** 26.5% (N=35) | **Study Aim:** To assess the effects of intensive nurse-led follow-up on self-management and compliance behaviors after myocardial infarction.  **Study design:** Multicenter, quasi-experimental, pre-post design without a control group  **Provider Involved:** Primary care nurses  **Intervention Delivery Platform:** Nursing consultation and self-care notebook | **Intervention:** The organizational structure was nodal, consisting of three levels. The first level, the Technical Research Group (TRG), comprised the two principal investigators and two coordinators responsible for monitoring four nodes. The second level, the Clinical Research Group (CRG), comprised eight nurses, each responsible for another node made up of 7–8 nurses from the CCG.  In the last intervention, the final post-intervention assessment of all outcome indicators was carried out. To minimize any bias caused by feeling observed and evaluated, which could influence the final assessment, the nurse could not see the assessment or scores assigned to the indicators in the previous consultations.  **Duration:** 12-18 months  **Frequency:** 11 structured nursing consultations (every 15-45 days; 30-60 minutes each). | N/A | **Primary Finding:** A total of 132 patients completed the intervention. The indicators for each NOC outcome and the variations in scores before and after the intensive follow-up showed a statistically significant improvement (p-value = 0.000).  Compliance Behavior: Prescribed Diet (pre = 3.7; post = 4.1);  Compliance Behavior: Prescribed Activity (pre = 3.9; post = 4.3);  Compliance Behavior: Prescribed Medication (pre = 3.9; post = 4.7). | This study shows that intensive follow-up improves patient compliance with diet, activity, and medication. Future research should examine the long-term effects, identify key intervention components, consider patient differences, evaluate scalability and cost-effectiveness, and explore the psychological factors behind improved adherence. |
| **Ref:** (Mallow et al., 2018)  **Year:** 2018  **Country:** United States  **Setting:** a free primary care clinic serving low-income, uninsured or underinsured adults. | **Primary disease:** Multiple Chronic Conditions  **Chronic Conditions Included:** DM, HT, Obesity, Hyperlipidimia, Depression  **Sample size:** 30 participants only the intervention group  **Age (Mean):** 52 years (SD=10, range 29-64)  **Sex:** 70% (N= 21) | **Study Aim:** To evaluate the initial effectiveness of the mI SMART intervention in improving biophysical outcomes in rural adults with multiple chronic conditions  **Study design:** Prospective pre/post design  **Provider Involved:** Nurse Practitioners  **Intervention Delivery Platform:** mI SMART (web-based app) with Bluetooth-enabled self-monitoring devices and video consultations. | **Intervention:** Each consenting  the participant was given Bluetooth-enabled self-monitoring devices such as a scale, glucometer, blood pressure cuff, and a Nexus 7  tablet, and three months of internet data service. The participants kept the equipment after the study period. Each participant used the mI SMART platform for 12 weeks to obtain healthcare from their location instead of traveling to the clinic. The period of twelve  weeks was chosen to overcome the potential for Hawthorne effect, allowing participants to establish a routine of usual chronic condition monitoring and follow-up  During the initial enrollment visit, each participant was given in person verbal and hands on instruction by one NP on how to use the tablet, the mI SMART platform, the self-monitoring devices, and personalized expectations from their NP of how often to use the self-monitors. Each participant was given a written copy of the  instructions, recorded demonstrations were also available within mI SMART for the participants to view at any time, and contact information for study personal for live technical support. Study  staff was available to answer any questions before the participant returned home to begin their 12-week intervention. During the 12  weeks, each participant used the video conferencing system to see their NP. The times of the video visits were arranged by the patients and the NP at their mutual convenience and the patient's need for care. Education videos related to care of their specific chronic conditions and live video conferencing with a health educator via the mI SMART platform were provided. The content of the videos and education were dependent on the unique combination of  chronic conditions of the participant. Frequency of obtaining selfmonitoring  readings varied by participant based on NP recommendations.  Patients received individualized automated reminders  for using the self-monitoring devices and taking medications. All self-monitoring readings received automated immediate feedback through mI SMART, and critical self-monitoring values were reviewed by a registered nurse. Appropriate referral was given when necessary.  **Duration:** 12 weeks  **Frequency:** Personalized based on patient need. | N/A | **Primary Finding:** Thirty participants were majority female, white, married, high-school educated or less, earning less than $20,000 per annum, and had multiple chronic conditions. Pre-intervention glucose, systolic blood pressure, diastolic blood pressure, weight and Body Mass Index were all reduced after the 12-week intervention.  **Random blood glucose**: Decreased from 201.93 to 146.79 mmol/L (p < .001)  **Systolic BP**: Decreased from 134.24 to 118.93 mmHg (p < .001)  **Diastolic BP**: Decreased from 88.79 to 83.62 mmHg (p < .001)  **BMI**: Decreased from 36.77 to 35.05 (p = .04) | This study emphasizes the encouraging positive results of the mI SMART intervention in improving health outcomes for rural adults with multiple chronic conditions. Future research should examine the long-term effects of the intervention, evaluate its effectiveness in larger and more diverse populations, identify the most effective components, and explore how socioeconomic factors influence the outcomes. |
| **Ref:** (Markle-Reid et al., 2021)  **Year:** 2021    **Country:** Canada    **Setting:** three large academic hospitals. | **Primary disease:** Not disease-specific    **Chronic conditions included:** Multimorbidity (patients and depressive symptoms, aged 65 or older)    **Sample size:**  Total: 127  Intervention: 63  Control:64    **Age in years (Mean±SD):**  Intervention: 77 (SD not reported)  Control: 77 (SD not reported)    **Sex (% Female):**  Intervention: 61.7%  Control: 63.5% | **Study Aim:** To evaluate the effectiveness of a nurse-led hospital-to-home transitional care intervention on mental functioning (primary outcome), physical functioning, depressive  symptoms, anxiety, perceived social support, patient experience, and health service use costs in older adults with multimorbidity and depressive symptoms.    **Study Design:** Pragmatic multi-site randomized controlled trial.    **Provider Involved: A** Care Transition Coordinator  (CTC) who is a Registered Nurse (RN).  **Intervention Delivery Platform**:  Individually tailored care (home visits, telephone follow-up and navigation support system) | **Intervention Name:**  Community Assets Supporting  Transitions (CAST)  intervention    **Intervention Detail:**  The intervention consisted of in-home visits and telephone calls by the CTCs that main activities included 1) assessment of the health and social care needs 2) identifying and managing depressive symptoms and multimorbidity 3) medication management 4) providing problem-solving therapy 5) implementing social and behavioural activation 6) providing education 7) communicating alerts to primary care providers regarding the presence of depressive symptoms, dementia, delirium, suicidal ideation. And provided system navigation support that consisted of 1) identifying and addressing any risk factors for adverse events 2) arranging community services 3) facilitating communication 4) supporting linkages and referrals 5) developing an individualized patient-centred plan of care 6) identifying health care professionals and plan for regular communication.    **Duration:** 6 months    **Frequency:** Home visit once a  month or at least twice in 6  months, with an average of 1  hour per visit | Usual care without a nurse-led hospital-to-home transitional care intervention | **Primary findings:**   - No significant group differences were seen for the baseline to six-month change in mental functioning (mean difference: 1.09; 95% CI: -3.24–5.41, p-value 0.61).     **Other findings:**   - No significant group differences were seen for the baseline to six-month change in physical functioning (mean difference: -1.45; 95% CI: -4.96, 2.07, p-value 0.42), depressive symptoms, (mean difference: 0.80; 95% CI: -1.43–3.03, p-value 0.48), anxiety, (mean difference: 1.34; 95% CI: -0.25–2.92, p-value 0.10) perceived social support (mean difference: 2.95; 95% CI: -1.93-7.83, p-value 0.23), - Older adults in the intervention group reported receiving more information about health and social services (p = 0.03) compared with the usual care group. - There was no statistically significant difference between groups in the change in total costs (including or excluding hospital costs) from baseline to 6-months (p = 0.07)     **Measurement of primary finding:**  Mental functioning was measured using the Mental Component Score (MCS) score from the Veterans Rand 12-item health survey (VR-12). | This study demonstrated inconclusive results for mental and physical functioning improvements in one aspect of patient experience, and the potential for significant improvements in perceived social support. However, a nurse-led transitional care intervention is a beneficial program because transitional care is a comprehensive approach to managing an older adult's health during transitions between care settings. By bridging these gaps, transitional care helps seniors maintain their health, reduces the risks of complications, and eliminates unnecessary hospital readmissions. Future research is also needed to ensure adequate sample size, improve the reach of the intervention, and to understand the influence of these contextual factors on study outcomes to inform decisions about wider implementation of the intervention and the need for further research. |
| **Ref:** (Moreno-Chico et al., 2021)  **Year:** 2021  **Country:** Spain  **Setting:** A primary care  center in Terrassa (a town near Barcelona) | **Primary disease:** Not disease-specific    **Chronic conditions included:** diabetes mellitus 2, high blood pressure, dyslipidemia, chronic obstructive pulmonary disease, heart failure, rheumatoid arthritis, or chronic kidney disease, aged 18 or older    **Sample size:**  Total: 118  Intervention: 58  Control:60    **Age in years (Mean±SD):**  Intervention: (64.33 **±** 7.86)  Control: (66.79 **±** 7.95)    **Sex (% Female):**  Intervention: 28.8%  Control: 28.8% | **Study Aim:** To examine the effectiveness of a nurse‐led, face‐to‐face, individually tailored health coaching (HC)  intervention in improving patient activation and secondary outcomes (self‐efficacy, quality of life, anxiety and depression symptoms, medication adherence, hospitalization, and emergency visits) among primary care users with chronic conditions.    **Study Design:** A two‐group quasi‐experimental time series trial    **Provider Involved:** Primary care nurse trained coach  **Intervention Delivery Platform**: face‐to‐face, individually‐tailored health coaching (HC | **Intervention Name:** Individualized Health Coaching (HC)    **Intervention Detail:**  Face‐to‐face, individually‐tailored health coaching (HC) based on activation theory, goal setting, planning, and visual tools that include educational, behavioral, and affective dimensions.  **Duration:** 6 weeks and follow‐up at 6 and 12 months    **Frequency:** 4-6 weekly HC sessions | Usual primary care. | **Primary findings:**   - Patients who received health coaching (HC) had significantly higher patient activation scores after the intervention at 6 weeks (73.29 vs. 66.51, p = .006). - However, as in the bivariate analysis, none of the differences in activation scores between groups were significant at the other time points (6, 12 months).     **Other findings:**   - There were no significant changes in secondary outcomes such as quality of life, self-efficacy, anxiety and depression, medication adherence, perceived social support, hospitalization, and emergency visits.     **Measurement of primary finding:**  Patient activation was measured with the European Spanish PAM-13. | This study shows a health coaching may be an effective strategy for achieving short‐term improvements in activating primary care users with chronic conditions. Further studies are needed to better understand how HC can improve and sustain changes in activation, as well as in other health outcomes, and to determine the methodological approaches best suited to capturing its effects. |
| **Ref:** (Yang et al., 2022)  **Year:** 2022    **Country:** China    **Setting:** Three community health centers in Changsha, China. | **Primary disease:** Not disease-specific    **Chronic conditions included:**  Patients aged ≥60 years with ≥3 of 38 chronic conditions, prescribed ≥1 chronic medication for ≥3 months, non-adherent to treatment, and self-managing their medications.    **Sample size:**  Total: 136  Intervention: 67  Control: 69    **Age in years (Mean±SD):**  Intervention: 70.76 ± 7.49  Control: 72.67 ± 7.64    **Sex (% Female):**  Intervention: 68.7%  Control: 52.2% | **Study Aim:** To evaluate the effectiveness of a nurse-led medication self-management intervention on medication adherence and health outcomes in older people with multimorbidity.    **Study Design:** A single-blind, two-arm randomized controlled trial.    **Provider Involved:** General practitioners and health care teams (Community Nurses)  **Intervention Delivery Platform**: In-person follow-up was conducted through one-on-one visits at community health centers, along with follow-up via telephone calls. | **Intervention Name:** Self-management intervention program informed by the Information–Motivation–Behavioral Skills (IMB) model.    **Intervention Detail:** The intervention, delivered by community nurses, comprised three face-to-face educational sessions. The first session involved assessing participants’ deficiencies in medication self-management and providing them with accurate information. The second session utilized motivational interviewing techniques to address barriers to adherence. The third session focused on enhancing self-management skills and developing an individualized medication management plan. Additionally, participants received two follow-up telephone calls to support ongoing adherence and address any medication-related issues.    **Duration:** 6 weeks    **Frequency of Intervention:**  Three face-to-face sessions, each lasting 30–40 minutes, were scheduled at 7–10 day intervals over a 4-week period. Additionally, two follow-up phone calls, each lasting approximately 15 minutes, were conducted biweekly. | Participants in the control group received usual care, which was managed by community general practitioners. | **Primary findings:**   - The intervention group showed a significant improvement in medication adherence at T1 (B = 1.63, P = 0.034; Cohen’s d = 0.45) and a non-significant increase of 0.95 at T2 compared to the control group.     **Other findings:**   - The intervention group showed significant improvements over time in medication knowledge and self-efficacy at T1 and T2 (all p < 0.05). At T2, significant changes were observed in beliefs about medication necessity (B = 2.68, p = 0.002) and concerns (B = -2.44, p = 0.002). Beliefs about medication harm improved significantly at T1 (B = -1.83, p < 0.001) only. No significant changes were found for beliefs about overuse, social support, or medication skills. - The intervention effects on treatment experiences, quality of life, and health care utilization were assessed. GEE analysis showed significant group-by-time interactions only for satisfaction with medication convenience at T1 (B = 5.02, p = 0.005) and medication burden at T2 (B = -3.96, p = 0.004). No significant interactions were observed for other treatment experience measures, EQ-5D-5L index, self-rated health, or health care utilization at either time point.     **Measurement of primary finding:**  Medication adherence was assessed using the MARS-5 scale. | This study highlights the short-term effectiveness of a nurse-led medication self-management intervention in improving medication adherence among older adults with multimorbidity. However, the benefits were not sustained in the long term. Future research should consider extended or reinforced interventions with ongoing follow-up, incorporate objective measures of adherence, evaluate cost-effectiveness, and examine the intervention’s impact across diverse populations and healthcare settings to improve generalizability and long-term outcomes. |

**References**

Boult, C., Reider, L., Frey, K., Leff, B., Boyd, C. M., Wolff, J. L., . . . Scharfstein, D. (2008). Early effects of "Guided Care" on the quality of health care for multimorbid older persons: a cluster-randomized controlled trial. *J Gerontol A Biol Sci Med Sci*, *63*(3), 321-327. <https://doi.org/10.1093/gerona/63.3.321>

Brand, C. A., Jones, C. T., Lowe, A. J., Nielsen, D. A., Roberts, C. A., King, B. L., & Campbell, D. A. (2004). A transitional care service for elderly chronic disease patients at risk of readmission. *Aust Health Rev*, *28*(3), 275-284. <https://doi.org/10.1071/ah040275>

Calvo, E., Izquierdo, S., Castillo, R., César, E., Domene, G., Gómez, A. B., . . . Ariza-Solé, A. (2021). Can an individualized adherence education program delivered by nurses improve therapeutic adherence in elderly people with acute myocardial infarction?: A randomized controlled study. *Int J Nurs Stud*, *120*, 103975. <https://doi.org/10.1016/j.ijnurstu.2021.103975>

Chew, H. S. J., Sim, K. L. D., Choi, K. C., & Chair, S. Y. (2021). Effectiveness of a nurse-led temporal self-regulation theory-based program on heart failure self-care: A randomized controlled trial. *International Journal of Nursing Studies*, *115*, 103872. <https://doi.org/https://doi.org/10.1016/j.ijnurstu.2021.103872>

Chow, S. K., & Wong, F. K. (2014). A randomized controlled trial of a nurse-led case management programme for hospital-discharged older adults with co-morbidities. *J Adv Nurs*, *70*(10), 2257-2271. <https://doi.org/10.1111/jan.12375>

Gräs Højgaard, H., Høgh, A. L., Lindholt, J. S., Frederiksen, K., & Dahl, M. (2025). Effect of nurse-led telephone follow-up to optimize adherence to preventive medication after screen-detected cardiovascular disease: A randomized controlled trial. *Eur J Cardiovasc Nurs*. <https://doi.org/10.1093/eurjcn/zvaf047>

Kashyap, N., Kavita, K., Saini, S., & Singh, A. (2022). Task sharing for managing common noncommunicable disease in a nurse led noncommunicable diseases clinic in peri-urban community of Chandigarh. *Indian Journal of Community Medicine*, *47*(4), 596-599. <https://doi.org/https://doi.org/10.4103/ijcm.ijcm_1048_21>

Liang, H. Y. P. R. N., Lin, L. H. M. M. D., Chang, C. Y. R. N., Wu, F. M. B. S. N. R. N., & Yu, S. P. R. N. (2021). Effectiveness of a Nurse-Led Tele-Homecare Program for Patients With Multiple Chronic Illnesses and a High Risk for Readmission: A Randomized Controlled Trial. *JOURNAL OF NURSING SCHOLARSHIP*, *53*(2), 161-170. <https://doi.org/https://doi.org/10.1111/jnu.12622>

Lizcano-Álvarez, Á., Carretero-Julián, L., Talavera-Saez, A., Cristóbal-Zárate, B., Cid-Expósito, M.-G., & Alameda-Cuesta, A. (2023). Intensive nurse-led follow-up in primary care to improve self-management and compliance behaviour after myocardial infarction. *Nurs Open*, *10*(8), 5211-5224. <https://doi.org/https://doi.org/10.1002/nop2.1758>

Mallow, J. A., Theeke, L. A., Theeke, E., & Mallow, B. K. (2018). The effectiveness of mI SMART: A nurse practitioner led technology intervention for multiple chronic conditions in primary care. *International Journal of Nursing Sciences*, *5*(2), 131-137. <https://doi.org/https://doi.org/10.1016/j.ijnss.2018.03.009>

Markle-Reid, M., McAiney, C., Fisher, K., Ganann, R., Gauthier, A. P., Heald-Taylor, G., . . . Whitmore, C. (2021). Effectiveness of a nurse-led hospital-to-home transitional care intervention for older adults with multimorbidity and depressive symptoms: A pragmatic randomized controlled trial. *PloS one*, *16*(7). <https://doi.org/https://doi.org/10.1371/journal.pone.0254573>

Moreno-Chico, C., Roy, C., Monforte-Royo, C., González-De Paz, L., Navarro-Rubio, M. D., & Gallart Fernández-Puebla, A. (2021). Effectiveness of a nurse-led, face-to-face health coaching intervention in enhancing activation and secondary outcomes of primary care users with chronic conditions. *Res Nurs Health*, *44*(3), 458-472. <https://doi.org/10.1002/nur.22132>

Tian, M., Wang, Y., Liu, J., & Wang, A. (2021). Clinical Study of Influence of Continuous Nursing Intervention Combined with Comfort Nursing Intervention under Medical-Nursing Combination on Self-Care Ability and Satisfaction of Elderly Patients with Chronic Diseases. *Evidence - Based Complementary and Alternative Medicine*, *2021*. <https://doi.org/https://doi.org/10.1155/2021/1464707>

Yang, C., Lee, D. T. F., Wang, X., & Chair, S. Y. (2022). Effects of a nurse-led medication self-management intervention on medication adherence and health outcomes in older people with multimorbidity: A randomised controlled trial. *International Journal of Nursing Studies*, *134*, 104314. <https://doi.org/https://doi.org/10.1016/j.ijnurstu.2022.104314>
